# Novel Effector RHIFs Identified From *Acidovorax avenae* Strains N1141 and K1 Play Different Roles in Host and Non-host Plants

**DOI:** 10.3389/fpls.2021.716738

**Published:** 2021-08-06

**Authors:** Minami Nakamura, Machiko Kondo, Aika Suzuki, Hiroyuki Hirai, Fang-Sik Che

**Affiliations:** ^1^Graduate School of Biosciences, Nagahama Institute of Bio-Science and Technology, Nagahama, Japan; ^2^Department of Bio-Science, Nagahama Institute of Bio-Science and Technology, Nagahama, Japan; ^3^Genome Editing Research Institute, Nagahama Institute of Bio-Science and Technology, Nagahama, Japan

**Keywords:** pathogen effector, hypersensitive response cell death, effector-triggered immunity, symptom formation, p-loop NTPase, rice, finger millet

## Abstract

Plant pathogenic bacteria inject effectors into plant cells using type III secretion systems (T3SS) to evade plant immune systems and facilitate infection. In contrast, plants have evolved defense systems called effector-triggered immunity (ETI) that can detect such effectors during co-evolution with pathogens. The rice-avirulent strain N1141 of the bacterial pathogen *Acidovorax avenae* causes rice ETI, including hypersensitive response (HR) cell death in a T3SS-dependent manner, suggesting that strain N1141 expresses an ETI-inducing effector. By screening 6,200 transposon-tagged N1141 mutants based on their ability to induce HR cell death, we identified 17 mutants lacking this ability. Sequence analysis and T3SS-mediated intracellular transport showed that a protein called rice HR cell death inducing factor (RHIF) is a candidate effector protein that causes HR cell death in rice. *RHIF*-disrupted N1141 lacks the ability to induce HR cell death, whereas *RHIF* expression in this mutant complemented this ability. In contrast, RHIF from rice-virulent strain K1 functions as an ETI inducer in the non-host plant finger millet. Furthermore, inoculation of rice and finger millet with either RHIF-deficient N1141 or K1 strains showed that a deficiency of *RHIF* genes in both strains results in decreased infectivity toward each the host plants. Collectively, novel effector RHIFs identified from *A. avenae* strains N1141 and K1 function in establishing infection in host plants and in ETI induction in non-host plants.

## Introduction

Plant pathogens employ sophisticated strategies to both evade plant immune systems and facilitate infection. In particular, plant pathogenic bacteria utilize effector proteins injected into plant cells using type III secretion systems (T3SS) as a means to circumvent plant defense mechanisms (Hueck, 1998; Buttner, 2016; Dey et al., 2019). When translocated into plant cells, these effectors alter various plant cell functions and promote successful infection by making the host more susceptible (Toruno et al., 2016). To combat such effectors, plants have evolved intracellular sensing systems to detect pathogen effectors either directly or indirectly while co-evolving with plant pathogens (Jones et al., 2016; Saur et al., 2020). Plant recognition of bacterial effector proteins is the basis for effector-triggered immunity (ETI; Jones and Dangl, 2006; Cui et al., 2015; Laflamme et al., 2020). Effector-triggered immunity is generally associated with strong immune responses, including hypersensitive response (HR) cell death which is one of the programmed cell death (Mur et al., 2008; Balint-Kurti, 2019; Bi and Zhou, 2021). Effector-triggered immunity induction is a critical determinant of host specificity of pathogenic bacteria because ETI is triggered by detecting molecules that pathogenic bacteria use to infect plants.

Several bacterial pathogen effectors have been demonstrated to adversely affect various host cellular processes. For example, *Pseudomonas syringae* HopX1 disrupts the jasmonate (JA) signaling by degrading the protein jasmonate zim domain (JAZ) (Gimenez-Ibanez et al., 2014). Transcriptional activator-like effectors produced by the rice (*Oryza sativa*) pathogen *Xanthomonas oryzae*, namely, PthXo1 and AvrXa7, promote sugar efflux from host plant cells by inducing the expression of several genes that encode SWEET family sugar transporters (Streubel et al., 2013; Macho, 2016). AvrRpt2, HopQ1, and AvrPtoB from *P. syringae* can sensitize host plant cells to the auxin, cytokinin, and abscisic acid signaling pathways, respectively, while AvrBs3 and AvrXccC from *Xanthomonas campestris* can induce several plant hormone signaling (Marois et al., 2002; Torres-Zabala et al., 2007; Cui et al., 2013; Ho et al., 2013; Hann et al., 2014). The *P. syringae* T3SS effector HopZ1 promotes infection by suppressing the isoflavone biosynthetic pathway (Zhou et al., 2011) while the shikimate and phenylpropanoid pathways are upregulated by WtsE from *Pantoea stewartii* upregulates to promote pathogen virulence (Asselin et al., 2015).

Pathogen effectors are secreted by plant pathogenic bacteria as well as animal pathogenic bacteria. However, plant pathogenic bacteria secrete 20–40 effectors during infection compared with the handful of effectors that animal pathogenic bacteria inject into animal cells (Macho, 2016). Co-evolution between plants and plant pathogens over millions of years has resulted in plants expressing many immune receptors as well as toxins to defend against infection (Frantzeskakis et al., 2019; Vries et al., 2020). Hence, plant pathogens employ a wide range of effectors to disable plant defenses, such as by nullifying toxins and disabling ETI induction (Block et al., 2013; Hurley et al., 2014). Individual effectors may employ diverse functional mechanisms, including yet unknown ones, in host vs. non-host plants.

Plant pathogen effectors are, in general, injected into plant cells using T3SS (Buttner, 2016). Type III secretion systems components are encoded by genes located in *hrp* (hypersensitive response and pathogenicity) gene clusters, and these genes within *hrp* operons are activated by two regulatory genes that are members of the OmpR family of two-component response regulators (Noel et al., 2002). We previously reported that the plant pathogen *Acidovorax avenae* possesses a 35-kb *hrp* gene cluster containing genes encoding T3SS components (Kondo et al., 2012). *Acidovorax avenae* is a Gram-negative bacterium that causes brown striped symptoms on the leaf sheaths of infected plants (Kadota et al., 1991). *Acidovorax avenae* has a wide host range among monocotyledonous plants, but individual strains of this pathogen infect only one or a few host species (Kadota et al., 1996). For example, strains isolated from rice, including K1, can infect only rice plants (rice-virulent), while strain N1141 isolated from finger millet (*Eleusine coracana*) cannot infect rice, even after being inoculated into rice tissues (rice-avirulent; Kadota et al., 1996). We reported that inoculation of rice with the rice-avirulent strain N1141 of *A. avenae* caused ETI responses, including HR cell death, accompanied by loss of plasma membrane permeability, nuclear DNA fragmentation, and upregulation of ETI-related genes, including *OsNAC4*, which positively regulates HR cell death (Che et al., 1999; Kaneda et al., 2009). In contrast, the rice-virulent strain K1 can suppress PAMP-triggered immunity (PTI) caused by N1141 flagellin but cannot induce ETI responses in rice (Kawaguchi et al., 2021). Interestingly, a T3SS-deleted N1141 mutant (*NΔT3SS*) did not cause ETI responses, while the T3SS-deleted K1 mutant (*KΔT3SS*) was defective in PTI suppression (Kondo et al., 2012; Kawaguchi et al., 2021). These results indicate that strains N1141 and K1 have multiple effectors with different functions in rice.

To determine how *A. avenae* effectors manipulate plant cell processes, we began by identifying PTI-suppressing effectors expressed by strain K1. The screening of transposon-tagged mutants based on PTI suppression ability, sequence analysis, transcriptome analysis, and *in silico* prediction of T3SS-mediated secretion showed that *A. avenae* K1 suppression factor 1 (AKSF1) is a candidate PTI suppressor. AKSF1 protein translocation into rice cells is dependent on T3SS during infection; furthermore, an *AKSF1*-disruption mutant lost its ability to suppress PTI responses while AKSF1 expression in the *AKSF1*-disruption mutant rescues the loss of PTI suppression. When rice plants were inoculated with *AKSF1*-disruption mutants, disease symptom severity was reduced and bacterial growth was suppressed. These results indicate that AKSF1 is a novel effector that can suppress PTI in host rice plants (Kawaguchi et al., 2021). Interestingly, whole-genome sequencing of strain N1141 revealed that it lacks *AKSF1*. Since the strict host specificity between *A. avenae* strains N1141 and K1 involves the T3SS-secreted effectors, AKSF1 is not a determinant of host specificity. To elucidate the mechanism underlying host specificity, novel effector molecules involved in determining host specificity must be identified.

In this study, we found that HR cell death was accompanied by the loss of plasma membrane integrity and nuclear DNA fragmentation in cultured rice cells and rice leaf sections following inoculation with strain N1141 in a T3SS-dependent manner. We screened transposon-tagged N1141 mutants based on their ability to induce HR cell death. Sequence analysis revealed that RHIF is a candidate ETI effector. *RHIF* gene disruption caused the loss of HR cell death induction ability while introduction of RHIF into an RHIF-deleted N1141 mutant complemented the induction ability of HR cell death. RHIF transport into rice cells and localization studies using a CyaA–RHIF fusion showed that RHIF is secreted through T3SS and localizes to the cytoplasm of rice cells. Interestingly, K1 RHIF with 13 amino acid substitutions can induce ETI responses, including HR cell death, in the non-host plant finger millet, but not in the host plant rice. Inoculation tests using RHIF-deficient strains N1141 and K1 on rice and finger millet showed that a deficiency of *RHIF* genes in strains N1141 and K1 results in decreased infectivity toward host plants. Our results therefore indicate that RHIFs isolated from strains N1141 and K1 are novel effectors that induce ETI in non-host plants and promote infection in host plants.

## Materials and Methods

### Plants and Bacteria

Suspension cultures of rice cells (line Oc) were provided from NAIST. Cultures of rice cells were grown at 30°C under light irradiation (Baba et al., 1986). The cells were diluted in fresh R2S medium (39.6 mm KNO_3_, 2.5 mm (NH_4_)_2_SO_4_, 1.0 mm MgSO_4_•7H_2_O, 1.0 mm CaCl_2_•2H_2_O, 1.7 mm NaH_2_PO_4_•2H_2_O, 20.1 μm EDTA•2Na, 19.8 μm FeSO_4_•7H_2_O, 6.6 μm MnSO_4_•4H_2_O, 7.7 μm ZnSO_4_•4H_2_O, 0.5 μm CuSO_4_•5H_2_O, 48.5 μm H_3_BO_3_, 0.6 μm NaMoO_4_•2H_2_O, 1% [w/v] Murashige and Skoog Vitamin powder (Sigma-Aldrich, St. Louis, United States), 18 μm 2,4-dichlorophenoxyacetic acid, and 87.6 mm Sucrose) every week, and all experiments were performed 4 days after transfer. *Acidovorax avenae* N1141 (MAFF 301141), T3SS-deficient N1141 strain (*NΔT3SS*), K1 (MAFF 301755), and T3SS-deficient K1 strain (*KΔT3SS*) were used as previously described (Kadota et al., 1996; Che et al., 2000; Kondo et al., 2017). Rice (*O. sativa L. subsp*. Kinmaze) was provided from NAIST. Finger millet (*E. coracana*) was purchased from TAKII & Co., Ltd. Rice and finger millet were grown for 3 weeks in a growth chamber with a 16 h day (200 μE m^−2^ s^−1^ at 30°C) and 8 h night (28°C) cycle.

### Cell Death Detection in Cultured Rice Cells

Cell death in cultured rice cells was detected by previously described method (Che et al., 1999). Cultured rice cells were inoculated with each bacteria strain (10^8^ cfu/ml), and incubated at 30°C for several periods. The cultured rice cells were transferred in 24-well tissue culture plates, the medium was removed, and then 0.05% Evans blue was added. 15 min later, cultured rice cells were washed three times with water, and the Evans blue dye in rice cells was extracted with extraction buffer (50% methanol and 1% SDS) for 12 h at room temperature. The amount of the extracted dye was estimated by measuring the absorbance at 595 nm.

### Cell Death Detection in Leaf Sheath Sections

Leaf sheaths of the seedlings were cut into 100 μm section with a razor (FEATHER, Osaka, Japan). Plate stem sections were incubated at 30°C with each bacterial strain (10^9^ cfu/ml). The stem sections were stained with 0.1% Evans blue for 15 min then washed extensively with distilled water to remove excess dye and observed by microscopy (Axioskop 2 plus, Carl Zeiss, Germany) used 40× objective lens.

### TUNEL Staining

Terminal deoxynucleotidyl transferase-mediated dUTP nick-end labeling (TUNEL) staining was performed by a previously described method (Che et al., 1999) with several modifications. Cultured cells were washed three times with phosphate buffered salts (PBS) (pH 7.4) and fixed in 4% (v/v) paraformaldehyde in PBS at 25°C overnight. The cells were washed three times with PBS and digested with proteinase K (10 μg/ml) containing 10 mm Tris–HCl (pH 7.5) at 37°C for 30 min and washed three times with PBS. The cells were subjected to TUNEL for 1 h at 37°C using a DeadEnd^™^ Fluorometric TUNEL System (Promega, Madison, WI, United States). After washing with PBS, 4ʹ,6-diamidino-2-phynylindole was added to the cells (1 μg/ml) in 50 mm NaCl, 5 mm EDTA, and 10 mm Tris–HCl (pH 7.4). Stained cells were observed by the confocal laser scanning microscope (FV1000, OLYMPUS, Tokyo, Japan; OLYMPUS UPlanSApo 40× lens, scan speed 20 us/pixel, laser intensity 5%, HV 600 V, Gain 1×, Offset 0%, and pinhole diameter 300 μm). The number of TUNEL-positive nuclei and DAPI staining nuclei was determined by counting nuclei within 20 individual fields. Each determination was done with at least 100 nuclei in each of three independent experiments.

### Generation of the Transposon-Tagged N1141 Mutant and Sequence Analysis the Transposon Insertion Site

To generate the transposon-tagged N1141 mutant, EZ-Tn5^™^<KAN-2>Tnp Transposome^™^ (EPICENTER, Lucigen, United States) was electro-transformed into *A. avenae* N1141 strain. Electroporation was performed using a Gene Pulser (Bio-Rad) under the conditions of voltage 1.7 kV/cm, capacitance 25 μf, and cuvette resistance 200 Ω. In order to clarify the sequence of transposon – inserted site, DNA near the transposon was amplified by the Random Amplification of Transposon Ends (RATE) method using primer set (5ʹ- CGAACTTTTGCTGAGTTGAAGGACT -3ʹ and 5ʹ- GAGCAAGACGTTTCCCGTTGAATAT -3ʹ).

### RNA Isolation and qRT-PCR

Total RNA was isolated from cultured rice cells using the RNeasy plant mini kit (Qiagen, Hilden, Germany) with DNase digestion. qRT-PCR was performed on Opticon2 instrument (Bio-Rad, Hercules, CA, United States), using the GoTaq One-Step RT-qPCR kit (Promega) with the following *OsNAC4*-specific primers (F: 5ʹ- TCCTGCCACCACCATTCTGAGATG -3ʹ and R: 5ʹ- TTGCAGAATCATGCTTGCCAG -3ʹ) and *PAL*-specific primers (F: 5ʹ- ACATCGGCAAGCTCATGTTC -3ʹ and R: 5ʹ- CCCTTGAACCCGTAGTCCAA -3ʹ). The mRNA levels were normalized based on the reference gene, *actin1* (F: 5ʹ- TCCATCTTGGCATCTCTCAG -3ʹ and R: 5ʹ- TGGCTTAG-CATTCTTGGGTC -3ʹ).

### Generation of RHIF Mutants

DNA was isolated from each strain using a previously described protocol (Wilson, 1987). To generate RHIF-deletion mutants, upstream and downstream regions of *RHIF* were PCR amplified using two sets of specific primers (F: 5ʹ- CAGGTCGACTCTAGAGATCGCCATCACCGGAGCCT -3ʹ and R: 5ʹ- GCTGTCATGCGGACATGATCCCCCGGAGCGTAC -3ʹ for the upstream region of *RHIF*; F: 5ʹ- GATCATGTCCGCATGACAGCCCACGGCGATG -3ʹ and R: 5ʹ- CGGGGATCCTCTAGACGCTATTACGGCGCCGTGGC -3ʹ for the downstream region of *RHIF*), respectively. Each amplified PCR products were diluted and mixed equally, and then, the mixed PCR products were re-amplified using the upstream region of *RHIF* F primer and the downstream region of *RHIF* R primer. The PCR product was cloned into *pK18mobsacBTET* linearized by *Xba*I using the In-fusion cloning kit (Takara Bio, Shiga, Japan), and the resulting plasmid was designated *NΔRHIF/pK18mobsacBTET*. The *NΔRHIF/pK18mobsacBTET* was electro-transformed into *A. avenae* N1141 strain. Electroporation was performed using a Gene Pulser (Bio-Rad; voltage 1.7 kV/cm, capacitance 25 μf, and cuvette resistance 200 Ω). The bacterial cells were plated on LB agar plates (containing 20 μg/ml of tetracycline) and incubated at 30°C for 48 h. For second crossing-over event, the provided bacterial strains were incubated in sucrose selection media (Pseudomonas F liquid medium containing 1% [w/v] Bacto Tryptone (Thermo Fisher Scientific, Waltham, United States), 1% [w/v] Proteose Peptone No. 3 (Thermo Fisher Scientific), 0.15% [w/v] K_2_HPO_4_, 0.15% [w/v] MgSO_,_ and 26% sucrose) at 30°C for 72 h.

To generate *RHIF* complementary N1141 mutant (*NΔRHIFC*)*, RHIF* was PCR amplified using the upstream region of RHIF F primer and the downstream region of *RHIF* R primer. The PCR product was cloned into *pK18mobsacBKm* linearized by *Spe*I using the In-fusion cloning kit (Takara Bio, Shiga, Japan), and the resulting plasmid was designated *NΔRHIFC/pK18mobsacBKm*. The *NΔRHIFC/pK18mobsacBKm* was electro-transformed into *NΔRHIF*. The bacterial cells were plated on LB agar plates (containing 50 μg/ml of kanamycin) and incubated at 30°C for 48 h. For second crossing-over event, the bacterial strain was incubated in sucrose selection media (Pseudomonas F liquid medium containing 27% sucrose) at 30°C for 72 h. *RHIF*-deletion K1 mutant (*KΔRHIF*) and *RHIF* complementary K1 mutant (*KΔRHIFC*) were generated in the same methods.

### Adenylate Cyclase Translocation Assay

Promoter region of *flaA* (336 bp) containing additional 5ʹ-terminal *XbaI* site and 3ʹ-terminal *Hin*dIII site was PCR amplified from *A. avenae* N1141 genome using specific primer set (5ʹ-TCTAGAGGCGGAACTCCTGCG-3ʹ and 5ʹ-AAGCTTCATTGCAAATCTCCTGAAAAGAAC-3ʹ) and cloned into *pGEM-T*. The plasmid was digested by *Xba*I and *Hin*dIII and cloned into *pBBR1Tp*, and the generated plasmid was named *flapro/pBBR1Tp*. *CyaA* was amplified with PCR using primer set (5ʹ-AAGCTTCAGCAATCGCATCAGGC-3ʹ and 5ʹ-GGTACCTCAGCTGTCATAGCCGGA-3ʹ) and cloned into *pGEM-T*. The plasmid was digested by *Hin*dIII and *Kpn*I and cloned into *flapro/pBBR1Tp*, and the generated plasmid was named *flapro-CyaA/pBBR1TP*. *RHIF* was PCR amplified from N1141 genome using specific primer set (5ʹ- AAGCTTATGTCCACTCCCCCTTCCCTGC -3ʹ and 5ʹ- AAGCTTTGCCCGCACCCCGAGC -3ʹ). The PCR product was cloned into *flapro-CyaA/pBBR1TP* linearized by *Hin*dIII using the In-fusion HD cloning kit (Takara Bio), and the generated plasmid, *flapro-RHIF-CyaA/pBBR1TP*, was transformed into N1141 or *NΔT3SS* by electroporation. Cultured rice cells were inoculated with 1.0 × 10^8^ cfu/ml of N1141 carrying *RHIF-CyaA* vector and incubated for 10 h at 30°C. After incubation, cultured rice cells were washed three times with distilled water and resuspended in 200 μl distilled water. The cells were boiled for 5 min, smashed with zirconia beads using the Bead Smash 12(WAKENYAKU, Kyoto, Japan) at 5,000 rpm for 5 min, and then centrifuged at 1,500 × *g* for 10 min at 4°C. Concentration of cAMP in the supernatant was measured using the Cyclic AMP EIA Kit (Cayman Chemical, Ann Arbor, MI, United States).

### Protoplast Preparation and Gene Transformation

Protoplast preparation and gene transformation were performed as described by Takai et al. (2007).

### Inoculation Test

Inoculation of *A. avenae* to rice or finger millet was performed by previously described method (Kondo et al., 2017), with the slightly modifications. The bacteria suspended in sterilized distilled water (1 × 10^6^ cfu/μl) were drop on the end of a needle in an amount of one microliter (10^6^ cfu), and then the sheath of 3 weeks old seedlings was pricked at a point 3 cm above the soil level. Inoculated seedlings were grown for 4 days in a growth chamber with a 16 h day (200 μE m^−2^ s^−1^ at 30°C) and 8 h night (28°C) cycle. The pathogenicity of each strain was estimated by assessing the brown stripe development around the inoculation site.

The growth of each bacterial strain was evaluated by measuring the number of bacterial cells in whole plants. Three sets of randomly selected rice plants were harvested, rinsed thoroughly in 1% HClO, 70% ethanol, and sterile water, and homogenized in distilled water. Dilutions of the homogenate were plated into Pseudomonas F agar. After incubation at 30°C for 48 h, the number of colony-forming units was determined.

## Results

### Rice ETI Induction by the Rice-Avirulent *Acidovorax avenae* Strain N1141

To identify ETI effectors, we first attempted to characterize ETI responses induced by the rice-avirulent *A. avenae* strain N1141. The loss of plasma membrane integrity is an important feature of HR cell death. Evans blue staining, which is used to detect the loss of plasma membrane integrity, is typically used to quantify dead cells. Therefore, we first examined rice HR cell death induced by strain N1141 using Evans blue staining.

**Figure 1:**
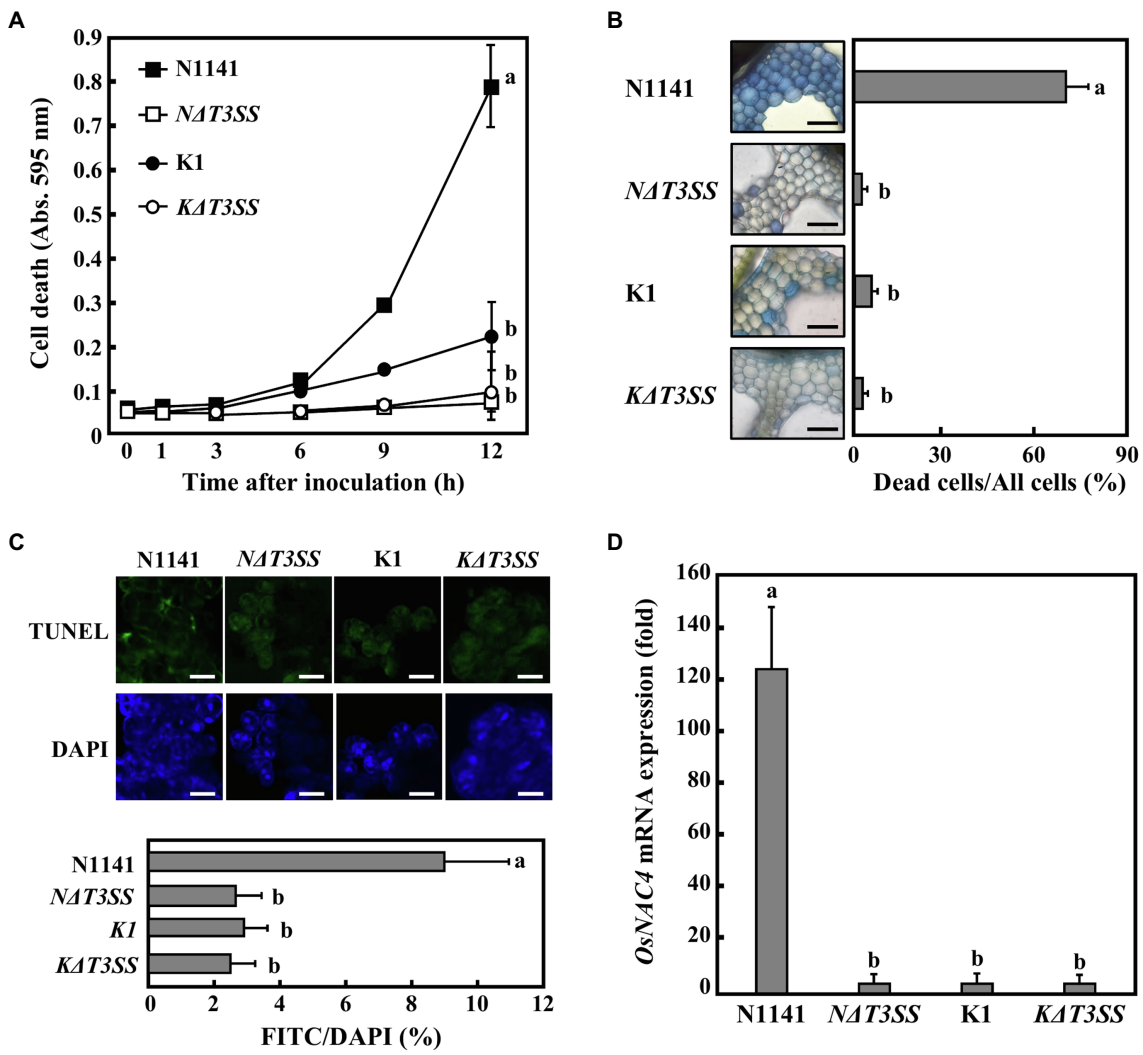
Rice hypersensitive response (HR) cell death induced by each strain of *Acidovolax. avenae* **(A)** Time course of HR cell death in cultured rice cells inoculated with N1141 wild type (solid square), *NΔT3SS* (open square), K1 wild type (solid circle), or *KΔT3SS* (open circle). Hypersensitive response cell death was detected by Evans blue staining. That time point values without letters indicate no statistical significance. **(B)** Induction of HR cell death in leaf sheath sections. Rice sheath cross-sections were inoculated with N1141, *NΔT3SS*, K1, and *KΔT3SS*, and 6 h after inoculation, Evans blue staining was performed. Bar represents 100 μm. Left graph shows the percentage of dead cell in leaf sheath sections. Each determination was done with at least 100 cells in each experiment. **(C)** Terminal deoxynucleotidyl transferase-mediated dUTP nick-end labeling (TUNEL) staining of each strain-inoculated cultured rice cells. Upper photographs represent TUNEL staining cell images of cultured rice cells inoculated with N1141 wild type, *NΔT3SS*, K1 wild type, or *KΔT3SS*. Lower photographs represent DAPI staining cell images of cultured rice cells inoculated with N1141 wild type, *NΔT3SS*, K1 wild type, or *KΔT3SS*. Scale bar in photographs represents 10 μm. Lower graph represents percentage of TUNEL-positive nuclei in cultured rice cells inoculated with each strain. The percentage of TUNEL-positive nuclei was determined by counting nuclei within 10 individual fields. Each determination was done with at least 1,000 nuclei in each of three independent experiments. **(D)** *OsNAC4* mRNA levels in cultured rice cells inoculated with N1141, *NΔT3SS*, K1, or *KΔT3SS*. The x-axis represents the fold change in mRNA levels relative to those in cultured cells prior to treatment. Error bars in all figures represent standard deviation of three independent experiments. Values followed by a different letter in figures were significantly different according to Tukey-Kramer test (*p* < 0.05).

When strain N1141 (10^8^ cfu/ml) was incubated with cultured rice cells, cell death was detected 6 h post-inoculation, and the number of dead cells gradually increased until 12 h post-inoculation. In contrast, inoculation with the rice-virulent strain K1 did not cause cell death in cultured rice cells until 6 h post-inoculation (Figure 1A). Unlike wild-type strain K1, neither T3SS-deficient strain N1141 (*NΔT3SS*) nor T3SS-deficient strain K1 (*KΔT3SS*) could induce the death of cultured rice cells (Figure 1A).

Next, we determined whether HR cell death induced by strain N1141 is also observed in rice leaf sheath sections. Staining rice leaf sheath sections with Evans blue showed that many stained cells were interspersed with unstained cells 6 h post-inoculation with strain N1141. In contrast, no stained cells were observed after inoculation with the virulent K1, *KΔT3SS*, or *NΔT3SS* strains (Figure 1B). Stained cells were not detected in the mock-inoculated sheath section (data not shown), which suggests that the loss of plasma membrane integrity observed in both cultured rice cells and rice sheath sections is induced by strain N1141, but not strain K1. Moreover, this induction is dependent on the T3SS of strain N1141.

Another important feature of HR cell death is nuclear DNA cleavage at specific chromosomal sites by DNA endonucleases (Ryerson and Heath, 1996; Ootsubo et al., 2016). To determine whether DNA fragmentation is associated with the loss of plasma membrane integrity induced by strain N1141 and whether the T3SS of N1141 is involved in DNA fragmentation, TUNEL (McCabe et al., 1997) was performed on cultured rice cells inoculated with either N1141, K1, or a T3SS-deficient strain. Terminal deoxynucleotidyl transferase-mediated dUTP nick-end labeling staining of cultured rice cells 6 h post-inoculation revealed that many fluorescein-derived bright green fluorescence signals were observed in N1141-inoculated cultured rice cells. The positions of all bright green signals coincided with those of nuclei stained with 4ʹ,6-diamidino-2-phenylindole (DAPI; Figure 1C). Terminal deoxynucleotidyl transferase-mediated dUTP nick-end labeling-positive nuclei were observed in 9% of N1141-inoculated cells. In contrast, the percentage of cultured rice cells inoculated with either K1, *KΔT3SS*, or *NΔT3SS* that showed TUNEL-positive nuclei was about 3%, lower than for N1141-inoculated cells (Figure 1C).

OsNAC4 is a plant-specific transcription factor that contains a consensus sequence called the NAC domain. We previously reported that OsNAC4 positively regulates HR cell death in rice. We therefore measured changes in OsNAC4 expression in cultured rice cells inoculated with either strain K1, *KΔT3SS*, or *NΔT3SS* using qRT-PCR. *OsNAC4* transcription was detected 6 h post-inoculation with N1141, but no induction of *OsNAC4* genes was observed in rice cells inoculated with either K1, *KΔT3SS*, or *NΔT3SS* (Figure 1D). Induction of *OsNAC4* expression during HR cell death indicates that *OsNAC4* expression can act as a marker for ETI induction. Taken together, our results suggest that the HR cell death specifically induced by strain N1141 is caused by effector proteins secreted through its T3SS.

### Identification of ETI Effector Genes From Strain N1141

To identify effector proteins that positively regulate HR cell death, a tagged transposon library was constructed for strain N1141. The workflow for high-throughput screening of the HR-inducing effector-encoding genes is described in Supplementary Figure S1. By screening 6,200 mutants according to this workflow, we found that 17 transposon-tagged N1141 mutants lost their ETI induction ability. Sequence analysis of transposon flanking regions using RATE polymerase chain reaction (Ducey and Dyer, 2002) revealed that the transposons were inserted into five unique genes, namely, *NT1, NT2, NT3, NT4*, and *NT5*.

**Figure 2:**
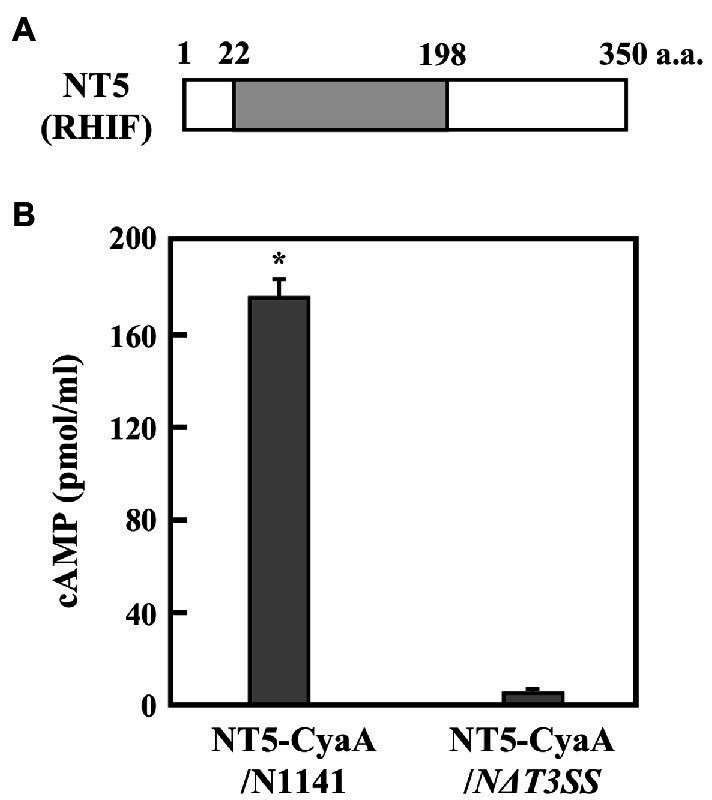
ETI effector candidate protein NT5 (RHIF). **(A)** Schematic drawing of NT5 (RHIF) structure. Gray box indicates the region that is homologous to the p-loop NTPase domain within rice isopentenyl transferase10. **(B)** Transport of NT5 (RHIF) from N1141 into rice cells. Cultured rice cells were inoculated with *A. avenae* N1141 strain and *N∆T3SS* carrying pBBR1Tp plasmid expressing the NT5 (RHIF)-CyaA fusion protein, and 12 h after inoculation, cAMP concentration was measured. Bars indicate the standard deviation of three independent experiments. Asterisk indicates significant difference (*t*-test, *p* < 0.05).

Among the five genes, *NT1, NT2*, and *NT3* are located within the *Hrp* cluster and encode HrpX, HrpD5, and HrcU, respectively. Additionally, *NT4* encodes a proton channel-related protein. Since the T3SS machinery includes these four proteins, *NT1–4* were not considered candidate effector genes because their deficiency would impair T3SS function. *NT5*, however, encodes an unrelated protein composed of 350 amino acids. A search of the GenBank/EMBL database for similar sequences identified a domain consisting of amino acid residues 22–198 within this protein that shows 43% identity to the P-loop NTPase domain of *O. sativa* (rice) isopentenyl transferase10 (Figure 2A).

If NT5 is an HR cell death inducible effector, then, it should be translocated into plant cells during infection. Adenylate cyclase (CyaA) requires calmodulin for its activity. Since calmodulin is only present in eukaryotic cells, CyaA is only active in eukaryotic cells (Sory and Cornelis, 1994). Thus, accumulation of the CyaA product, cAMP, in inoculated plant tissues can be used to measure type III effector delivery into plant cells (Casper-Lindley et al., 2002; Schechter et al., 2004). We monitored the intracellular transport of NT5 using an NT5-CyaA hybrid protein. In order to do this, we generated N1141 and *NΔT3SS* transformants that express NT5-CyaA. We extracted cAMP from cultured rice cells 9 h post-inoculation and measured its concentration using an enzyme immunoassay. High levels of cAMP (about 180 pmol/ml) were detected in cultured rice cells inoculated with N1141 expressing NT5-CyaA, whereas very small amounts of cAMP accumulation were observed in cells inoculated with *NΔT3SS* expressing NT5-CyaA (Figure 2B), suggesting that NT5 is transported into rice cells through the T3SS of strain N1141. Based on these data, we concluded that NT5 is a candidate effector protein that causes HR cell death in rice. We therefore named NT5 rice HR cell death inducing factor (RHIF).

### Identification of RHIF as an ETI Effector in Rice

**Figure 3:**
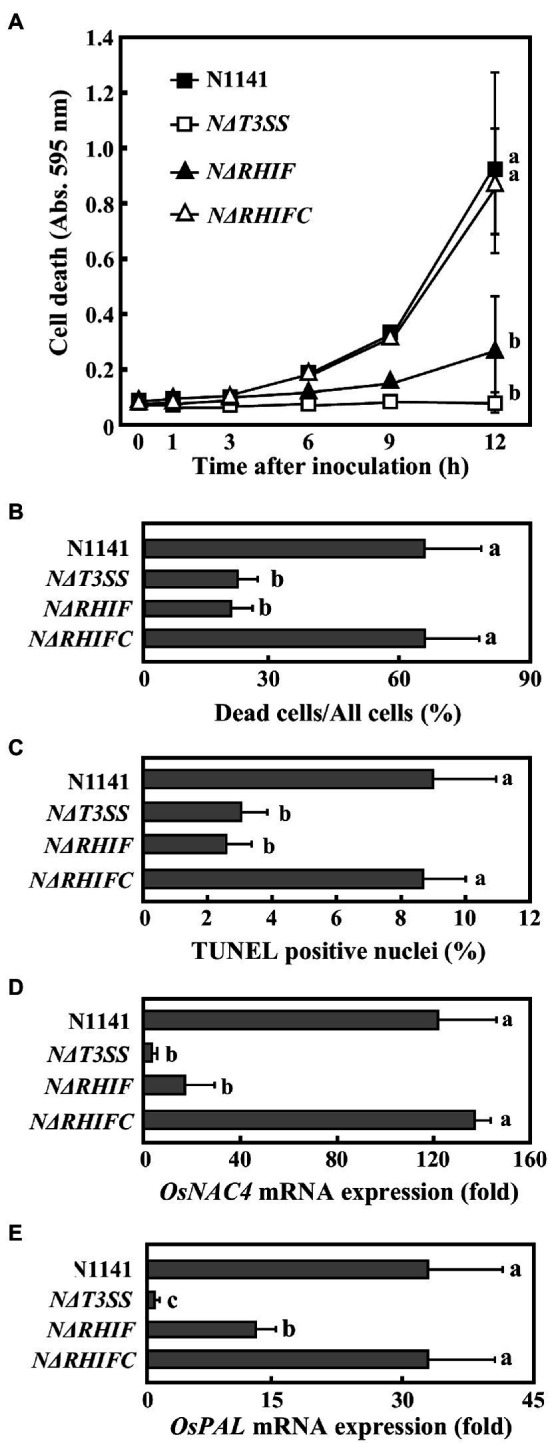
Role of RHIF in induction of rice ETI. **(A)** Time course of HR cell death in cultured rice cells inoculated with N1141 (solid square), *NΔT3SS* (open square), *NΔRHIF* (solid triangle), or *NΔRHIFC* (open triangle). That time point values without letters indicate no statistical significance. **(B)** Percentage of dead cell in leaf sheath sections. Rice sheath cross-sections 6 h after treatment with N1141, *NΔT3SS*, *NΔRHIF*, and *NΔRHIFC* were monitored for dead cells using Evans blue staining. Each determination was done with at least 100 cells in each experiment. **(C)** Percentage of TUNEL-positive nuclei in cultured rice cells inoculated with N1141, *NΔT3SS*, *NΔRHIF*, and *NΔRHIFC*. The percentage of TUNEL-positive nuclei was determined by counting nuclei within 10 individual fields. Each determination was done with at least 1,000 nuclei in each of three independent experiments. **(D)** *OsNAC4* mRNA levels in cultured rice cells inoculated with N1141, *NΔT3SS*, *NΔRHIF*, and *NΔRHIFC*. The x-axis represents the fold change in mRNA levels relative to those in cultured cells prior to treatment. **(E)** *PAL* mRNA levels in cultured rice cells inoculated with N1141, *NΔT3SS*, *NΔRHIF*, and *NΔRHIFC*. The x-axis represents the fold change in mRNA levels relative to those in cultured cells prior to treatment. Error bars in figures represent standard deviation of three independent experiments. Values followed by a different letter in figures were significantly different according to Tukey-Kramer test (*p* < 0.05).

To determine whether RHIF functions as an effector that causes HR cell death in rice, we generated an *RHIF*-deletion N1141 mutant (*NΔRHIF*). We also generated an RHIF-complement *NΔRHIF* mutant (reintroduction of *RHIF* into the genome of *NΔRHIF: NΔRHIFC* mutant) using homologous recombination. Cultured rice cells were inoculated with either strain N1141, *NΔRHIF, NΔRHIFC*, or *NΔT3SS*. Plasma membrane integrity was monitored by Evans blue staining. Clear cell death was induced in N1141-inoculated cultured cells 6 h post-inoculation, while no cell death was observed in cultured rice cells inoculated with *NΔRHIF* (Figure 3A). When cultured rice cells were inoculated with *NΔRHIFC*, which is the RHIF-complement *NΔRHIF*, cell death was induced to the same extent as that observed in N1141-inoculated cultured rice cells (Figure 3A). Similarly, Evans blue staining showed that cell death in rice leaf sheaths was induced by inoculation with *NΔRHIFC*, but not by *NΔRHIF* (Figure 3B).

We used a TUNEL assay to determine whether the DNA fragmentation observed during HR cell death was also induced by RHIF. Cultured rice cells were inoculated with N1141, *NΔRHIF*, *NΔRHIFC*, and *NΔT3SS*. Six hours post-inoculation, TUNEL was performed. The percentage of TUNEL-positive nuclei observed in N1141- and *NΔRHIFC*-inoculated cultured rice cells reached about 8%, while the percentage of TUNEL-positive nuclei in the cultured rice cells inoculated with *NΔRHIF* and *NΔT3SS* was only about 3% (Figure 3C).

To clarify the involvement of RHIF in the induction of *OsNAC4*, which is an indicator of ETI induction, *OsNAC4* mRNAs were quantified in N1141-, *NΔRHIF*-, *NΔRHIFC*-, and *NΔT3SS*-inoculated cultured rice cells. The *OsNAC4* expression level was about 100 times higher in cultured rice cells inoculated with either N1141 or *NΔRHIFC* than in cells inoculated with either *NΔRHIF* or *NΔT3SS* (Figure 3D).

It was reported that ETI and HR can sometimes be uncoupled (Chiang and Coaker 2015; Menna et al., 2015). We previously reported that *OsPAL* which is involved in plant secondary metabolism is T3SS-dependently induced by N1141 of *A. avenae* (Kondo et al., 2012). To determine whether RHIF positively regulates ETI as well as HR cell death, *OsPAL* mRNA were quantified in N1141-, *NΔRHIF*-, *NΔRHIFC*-, and *NΔT3SS*-inoculated cultured rice cells (Figure 3E). 6 h after inoculation, the expression level of *OsPAL* was about 30 times higher in cultured rice cells inoculated with either N1141 or *NΔRHIFC* than in cells before inoculation. In contrast, *OsPAL* expression level in *NΔRHIF*-inoculated cultured rice cells was lower than that in N1141-ioculated cultured rice cells, and no increase of *OsPAL* expression was observed in *NΔT3SS*-inoculated cultured rice cells. These results indicate that RHIF from N1141 induces not only HR cell death but also ETI.

**Figure 4:**
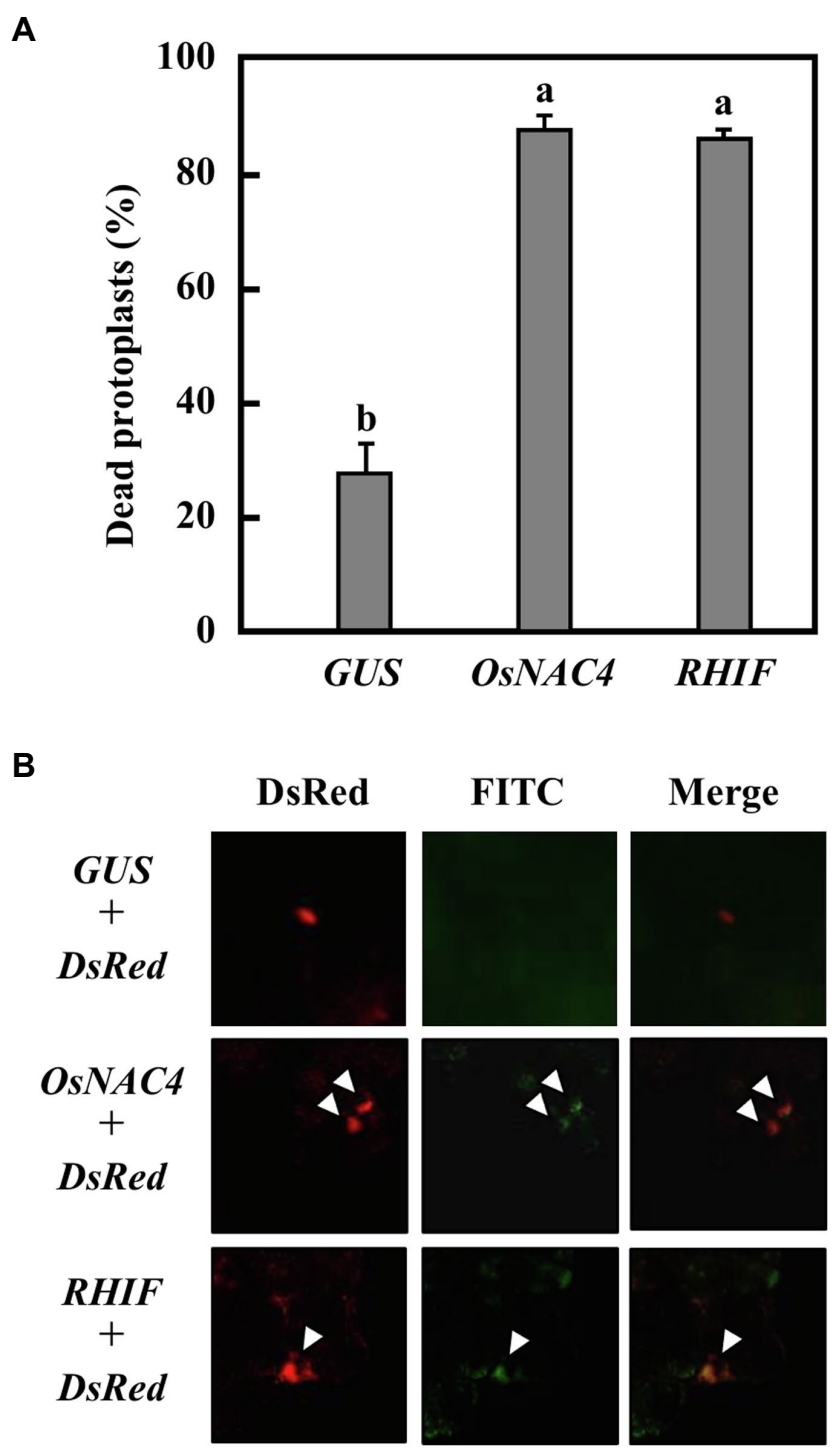
HR cell death induction by *RHIF*. **(A)** Percentage of dead protoplasts induced by expression *GUS*, *OsNAC4*, and *RHIF*. HR cell death was detected by Evans blue staining. Error bars represent standard deviation of three independent experiments. Values followed by a different letter were significantly different according to Tukey-Kramer test (*p* < 0.05). **(B)** DNA fragmentation detected by TUNEL staining. *GUS* and *DsRed* (upper panels), *NRHIF* and *DsRed* (middle panels), and *KRHIF* and *DsRed* (lower panels) were co-expressed. The arrow indicates the TUNEL-positive nucleus.

To determine whether HR cell death is triggered by RHIF transported into rice cells, we constructed three plasmids, one of which contains the *RHIF* gene while the others contain either *GUS* as a negative control or *OsNAC4* as a positive control. The individual plasmids were used to transform rice protoplasts. As shown in Figure 4A, approximately 80% of RHIF- and OsNAC4-expressing rice protoplasts were stained with Evans blue, while the proportion of dead cells in GUS-expressing rice protoplasts was significantly reduced. Because another important feature of rice HR cell death induced by strain N1141 is nuclear DNA fragmentation (Che et al., 1999), TUNEL staining was attempted on rice cells expressing RHIF. Gold particles for bombardment were coated with a mixture of *RHIF*-expressing vector and *DsRed*-expressing vector before being bombarded into cultured rice cells. Six hours post-inoculation, the *RHIF*-expressing cultured rice cells (DsRed-positive cells) exhibited fluorescein-derived bright green fluorescence of their nuclei, as detected by TUNEL (Figure 4B). Similar nuclear DNA fragmentation was also observed in OsNAC4-expressing cultured rice cells, while no fluorescein-derived bright green fluorescence was found in GUS-expressing cultured rice cells, suggesting that ectopic expression of RHIF in rice cells causes nuclear DNA fragmentation. Based on the above results, we concluded that HR cell death in rice is induced by RHIF transported from N1141 into rice cells.

### Role of RHIF Derived From Strain K1 in ETI Induction

**Figure 5:**
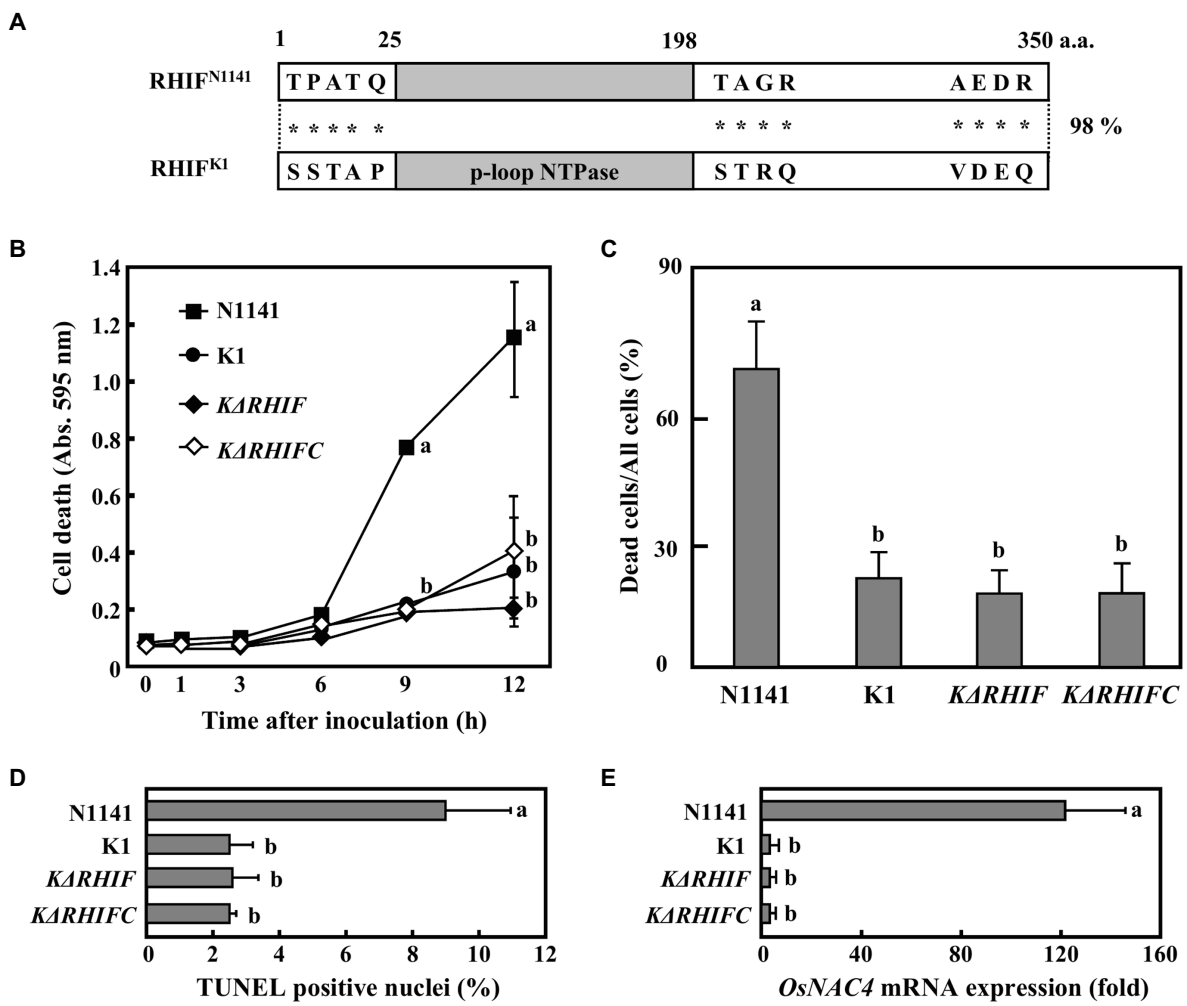
Role of KRHIF in induction of rice ETI. **(A)** Schematic drawing of RHIFs. Gray box indicates the p-loop NTPase domain. Asterisks indicate non-conserved amino acid residues. **(B)** Time course of HR cell death in cultured rice cells inoculated with N1141 (solid square), K1 (solid circle), *KΔRHIF* (solid rhomb), or *KΔRHIFC* (open rhomb). That time point values without letters indicate no statistical significance. **(C)** Percentage of dead cell in leaf sheath sections. Rice sheath cross-sections 6 h after treatment with N1141, K1, *KΔRHIF* and *KΔRHIFC* were monitored for dead cells by Evans blue staining. Each determination was done with at least 100 cells in each experiment. **(D)** Percentage of TUNEL-positive nuclei in cultured rice cells inoculated with N1141, K1, *KΔRHIF*, and *KΔRHIFC*. The percentage of TUNEL-positive nuclei was determined by counting nuclei within 10 individual fields. Each determination was done with at least 1,000 nuclei in each of three independent experiments. **(E)** *OsNAC4* mRNA levels in cultured rice cells inoculated with N1141, K1, *KΔRHIF*, and *KΔRHIFC*. The x-axis represents the fold change in mRNA levels relative to those in cultured cells prior to treatment. Error bars in figures represent standard deviation of three independent experiments. Values followed by a different letter in figures were significantly different according to Tukey-Kramer test (*p* < 0.05).

As mentioned above, strain N1141 causes rice ETI responses, but strain K1 does not. If strain K1 expresses RHIF, what role does K1-derived RHIF play in inducing ETI responses? Therefore, we next attempted to identify the *RHIF* gene of strain K1. *RHIF* gene derived from strain K1 was amplified by PCR using strain K1 genomic DNA as a template, and the sequence of the amplified DNA fragment was determined. We found that the putative *RHIF* gene contains an open reading frame of 1,050 bp, located at the same chromosomal locus as the N1141-derived *RHIF* gene. The translated amino acid sequence revealed that the RHIF derived from strain K1 is also composed of 350 amino acids, with a difference of only 13 residues compared with the N1141 ortholog (Figure 5A). Interestingly, all residues that differ between both RHIFs are present outside the P-loop NTPase domain (Figure 5A). Since strain K1 carries a different *RHIF* allele from that of strain N1141, we named the N1141 allele *NRHIF* and the K1 allele *KRHIF*.

To determine the role of KRHIF in ETI induction, we generated a *KRHIF*-deletion K1 mutant (*NΔRHIF*) and a *KRHIF* complementation *KΔRHIF* mutant (reintroduction of KRHIF into the genome of *KΔRHIF: KΔRHIFC*). Cultured rice cells were inoculated with either strain K1, *KΔRHIF*, or *KΔRHIFC*, and cell death was monitored by Evans blue staining. Cell death in cultured rice cells was not induced not only by K1 but also by *KΔRHIF* or *KΔRHIFC* until 12 h post-inoculation (Figure 5B). When rice leaf sheaths were inoculated with either K1, *KΔRHIF*, or *KΔRHIFC*, we did not detect cell death (Figure 5C). Moreover, we could not detect nuclear DNA fragmentation in cultured rice cells inoculated with either K1, *KΔRHIF*, or *KΔRHIFC* (Figure 5D). Similarly, the expression level of *OsNAC4* in cultured rice cells was not increased by inoculation with either *KΔRHIF* or *KΔRHIFC* (Figure 5E). Furthermore, when KRHIF was expressed in cultured rice cells, neither cell death nor nuclear DNA fragmentation was observed (Supplementary Figure S2). These results indicate that KRHIF does not function as an ETI-inducible effector in rice.

**Figure 6:**
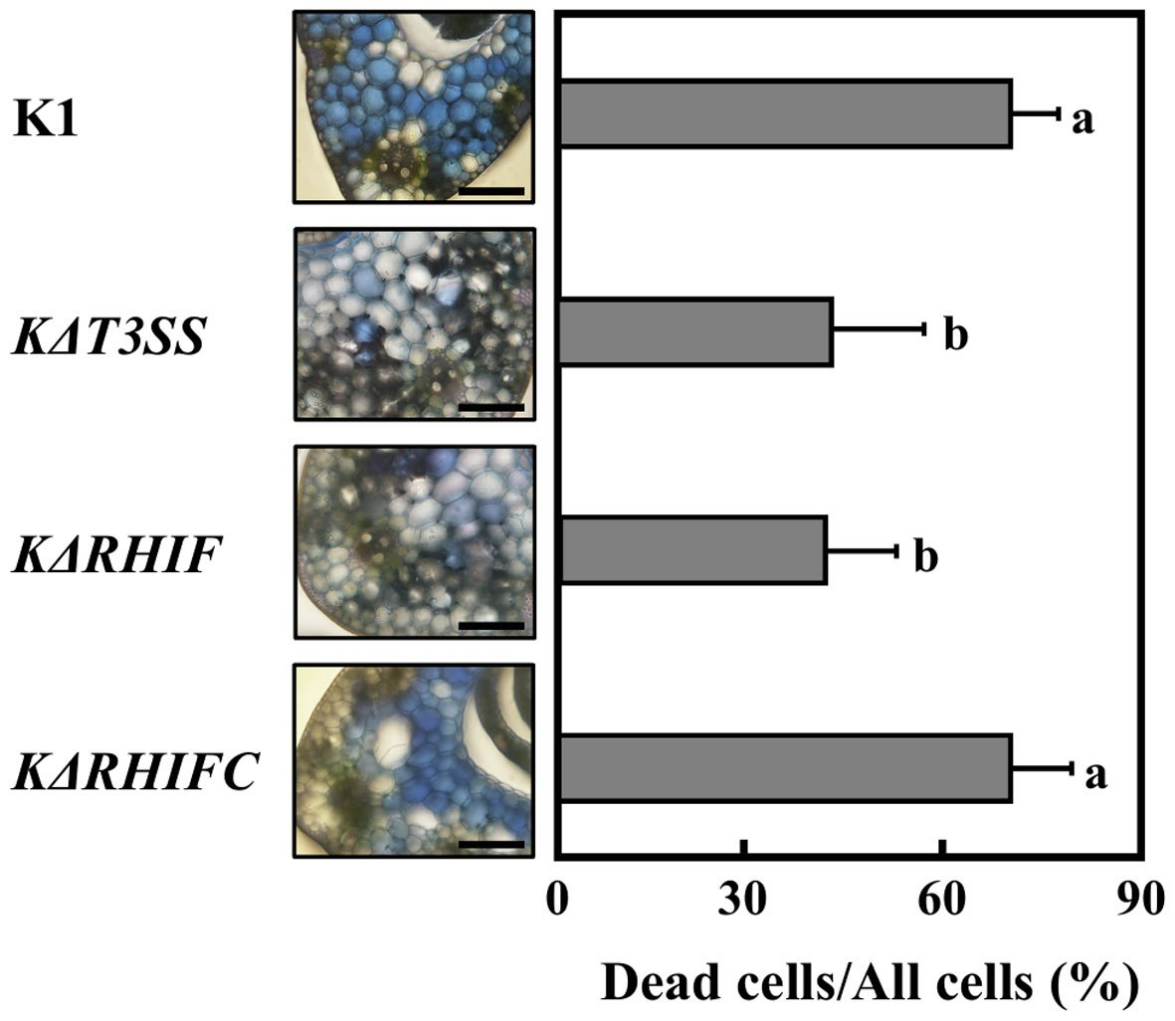
Role of KRHIF in induction of finger millet cell death. Finger millet sheath cross-sections were inoculated with K1, *KΔT3SS*, *KΔRHIF*, and *KΔRHIFC*, and 12 h after inoculation, Evans blue staining was performed. Scale bar represents 100 μm. Light graphs show the percentage of dead cell in leaf sheath sections of finger millet. Each determination was done with at least 100 cells in each experiment. Error bars represent standard deviation of three independent experiments. Values followed by a different letter were significantly different according to Tukey-Kramer test (*p* < 0.05).

The host plants of strains K1 and N1141 are rice and finger millet, respectively. Therefore, we next evaluated the effect of KRHIF on the non-host finger millet. Evans blue staining of finger millet leaf sheath sections inoculated with strain K1 showed that cell death was induced in more than 60% of cells 12 h post-inoculation. A reduction in dead cells was observed in sheath sections inoculated with either *KΔT3SS* or *KΔRHIF* (Figure 6). Cell death was induced to the same extent in finger millet leaf sheath sections at the same level by strain K1 and the KRHIF complementation mutant, *KΔRHIFC* (Figure 6), which suggests that *KRHIF* functions as an effector that induces cell death in finger millet.

### Roles of NRHIF and KRHIF in Disease Formation in Rice and Finger Millet

**Figure 7:**
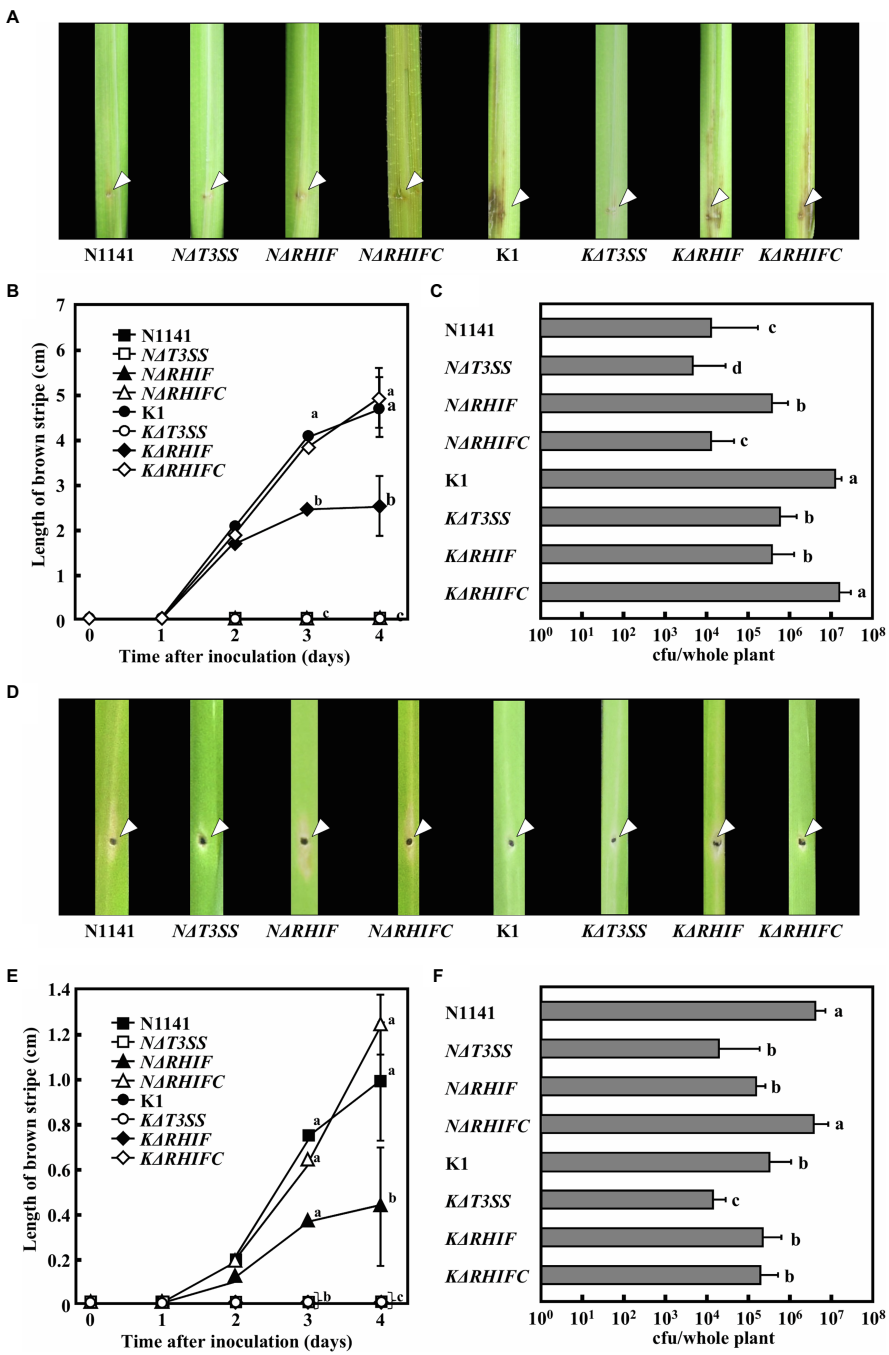
Inoculation tests of N1141, *NΔT3SS*, *NΔRHIF*, *NΔRHIFC*, K1, *KΔT3SS*, *KΔRHIF*, and *KΔRHIFC*. **(A)** Photographs of rice inoculated with each strain. Each strain was inoculated into rice using the single-needle method, and symptoms were observed 4 days later. The arrow indicates the inoculation points. **(B)** Length of brown stripe symptoms in N1141 (solid square)-, *NΔT3SS* (open square)-, *NΔRHIF* (solid triangle)-, *NΔRHIFC* (open triangle)-, K1 (solid circle)-, *KΔT3SS* (open circle)-, *KΔRHIF* (solid diamond)-, and *KΔRHIFC* (open diamond)-inoculated rices. That time point values without letters indicate no statistical significance. **(C)** Number of bacterial cells in whole rice plants 4 days after inoculation. Error bars indicate the standard deviation of three independent experiments. **(D)** Photographs of finger millet inoculated with each strain. Bacterial strains were inoculated into finger millet using the single-needle method, and symptoms were observed 4 days later. The arrow indicates the inoculation points. **(E)** Length of brown stripe symptoms in N1141 (solid square)-, *NΔT3SS* (open square)-, *NΔRHIF* (solid triangle)-, *NΔRHIFC* (open triangle)-, K1 (solid circle)-, *KΔT3SS* (open circle)-, *KΔRHIF* (solid diamond)-, and *KΔRHIFC* (open diamond)-inoculated finger millets. That time point values without letters indicate no statistical significance. **(F)** Number of bacterial cells in whole finger millet plants 4 days after inoculation. Bars indicate standard deviation of three independent experiments. Error bars in figures represent standard deviation of three independent experiments. Values followed by a different letter in figures were significantly different according to Tukey-Kramer test (*p* < 0.05).

To clarify the roles of NRHIF and KRHIF on disease formation in rice, rice plants were inoculated with either N1141, K1, *NΔT3SS*, *KΔT3SS*, *NΔRHIF*, *KΔRHIF, NΔRHIFC*, or *KΔRHIFC* using the needle injection method. Brown stripe symptoms in K1-inoculated rice appeared 2 days post-inoculation and gradually spread 4 days post-inoculation (Figures 7A,B). In contrast, when rice was inoculated with either N1141, *NΔRHIF*, *NΔRHIFC*, *NΔT3SS*, or *KΔT3SS*, no brown stripe symptoms appeared within 4 days post-inoculation (Figures 7A,B). Interestingly, inoculation with *KΔRHIF* reduced the severity of symptoms by about 50%; this reduction was restored by RHIF complementation (Figures 7A,B). The presence of inoculated bacteria was determined by measuring internal bacterial load. When wild-type K1 (1 × 10^6^ cfu) was inoculated into rice, the number of K1 cells reached 3 × 10^7^ cfu/plant 4 days post-inoculation (Figure 7C). No remarkable increase in the numbers of *KΔRHIF, KΔT3SS*, and *NΔRHIF* cells was observed 4 days post-inoculation (Figure 7C). In contrast, the numbers of N1141, *NΔT3SS*, and *NΔRHIFC* in rice 4 days post-inoculation was lower than the number of inoculated bacterial cells. These observations, together with the lesion formation data, indicate that the absence of *NRHIF* genes increases the infectivity of strain N1141, and the absence of the *KRHIF* gene decreases the infectivity of strain K1 toward rice.

To determine the role of NRHIF and KRHIF in disease formation in finger millet, we conducted a similar inoculation test on finger millet with N1141, K1, *NΔT3SS*, *KΔT3SS*, *NΔRHIF*, *KΔRHIF, NΔRHIFC*, and *KΔRHIF*. The brown stripe symptom in finger millet appeared 4 days post-inoculation with finger millet-virulent strain N1141. We observed a 30% reduction in symptoms in finger millet inoculated with *NΔRHIF* while the symptoms observed in finger millet inoculated with the RHIF complementation strain, *NΔRHIFC*, were similar to those inoculated with wild-type strain N1141 (Figures 7D,E). When the number of each bacterial strain in plants was measured 4 days post-inoculation, we found that the number of N1141 cells increased by about three times compared with the number of inoculated cells. In contrast, the number of bacteria decreased in finger millet inoculated with *NΔRHIF*, and *NΔRHIFC* proliferated in finger millet to the same level as wild-type N1141. Unexpectedly, RHIF-deficient strain K1 did not appear to proliferate in finger millet (Figure 7F). These results indicated that *NRHIF* deficiency decreases strain N1141’s infectivity toward finger millet.

## Discussion

In this study, we demonstrate that NRHIF identified from the rice-avirulent strain N1141 of *A. avenae* is novel effector that induces ETI including HR cell death in rice. In contrast, KRHIF from rice-virulent strain K1 functions as an ETI inducer in the non-host plant finger millet. Finally, we show that inoculation of rice and finger millet with either RHIF-deficient N1141 or K1 strains showed that a deficiency of *RHIF* genes in both strains results in decreased infectivity toward each host plants. To clarify that NRHIF and KRHIF play different roles for each host plant, the *NRHIF* and *KRHIF* genes-swapping mutants are useful. Therefore, we generate two swapping mutants; *KRHIF*/*NΔRHIF* strain in which *KRHIF* was introduced into *NΔRHIF* genome, *NRHIF*/*KΔRHIF* strain in which *NRHIF* was introduced into *KΔRHIF* genome. However, ETI induction ability and infectivity of each swapping mutant could not be evaluated because poor growth of the mutants, decreased expression and instability of RHIFs were observed in each swapping mutants. We conclude that NRHIF and KRHIF play different roles for each host or non-host plant from the collective of these experimental data.

The P-loop NTPase domain is common to both NRHIF and KRHIF. The P-loop NTPase domain is the most prevalent domain among bacterial and eukaryotic proteins (Leipe et al., 2003). While there is some variety in the enzyme-catalyzed reaction of the P-loop NTPase, the most common one is the hydrolysis of the β–γ phosphate bond of a bound nucleoside triphosphate (Arya and Acharya, 2018). The P-loop NTPase domain of NRHIF and KRHIF has high sequence homology with the P-loop NTPase domain of tRNA isopentenyltransferase that catalyzes the addition of the 5-carbon isopentenyl moiety to the exocyclic amine of adenine 37 located outside the anticodon using dimethylallyl pyrophosphate (DMAPP; Sakamoto et al., 2006). Detailed studies have been conducted on the structure–activity relationship of the *MiaA* encoded tRNA isopentenyltransferase. Gln166, which is present in the P-loop of MiaA, is conservatively replaced by bulky charged residues, while Arg167 is strictly conserved between species (Soderberg and Poulter, 2001). Substitution of Arg167 with Ala resulted in a significant reduction in catalytic efficiency, with *K*_m_ increased by 20 times for RNA and nearly 10 times for DMAPP compared to wild-type (Chimnaronk et al., 2009). The conservation of Glu187 and Arg188 within the P-loop NTPase domain of RHIFs indicates that the P-loop NTPase domain of RHIF retains its enzymatic activity. The P-loop NTPase domain is involved in a variety of complex biological processes, including programmed cell death, disease, and immune responses in plants and animals through its own enzymatic activity (Leipe et al., 2004). NRHIF and KRHIF caused ETI responses, including HR cell death in non-host plants. The 13 amino acid differences between NRHIF and KRHIF are also located outside the P-loop NTPase. Therefore, it is highly likely that the pleiotropic effects, including ETI responses and the development of symptoms triggered by KRHIF and NRHIF in host and non-host plants, are caused by the enzymatic activity of their P-loop NTPase domain.

The functional dualities of KRHIF and NRHIF may be due to the presence of specific *R*-like genes corresponding to KRHIF and NRHIF. Since there is an R-like protein corresponding to NRHIF in rice and a different R-like protein corresponding to KRHIF in finger millet, KRHIF and NRHIF induce different biological reactions in rice and finger millet. The specific correspondence between each RHIF and an appropriate R-like protein is likely defined by a 13 amino acid difference between the both RHIFs. Furthermore, these different amino acids also need to abolish the development of symptoms mediated by the P-loop NTPase activity of RHIF isolated from avirulent strains.

Recognition of its corresponding effector by an R protein initiates a defense signaling cascade that results in ETI, including HR cell death (Arya and Acharya, 2018). Many R proteins expressed by plants belong to the nucleotide-binding and leucine-rich repeat protein family (NB-LRR). The NB-LRR class of R proteins can be further divided into two groups based on whether they have a Toll/interleukin-1 receptor domain or a coiled-coil (CC) domain at their N-terminus (Collier and Moffett, 2009). Analysis of various plant genomes, including those of *A. thaliana*, *O. sativa*, *Solanum tuberosum* (potato), *Brassica rapa* (field mustard), and *Brachypodium distachyon* (purple false brome), has demonstrated that the NBS-LRR class of R proteins is among the largest gene families in Kingdom Plantae (Marone et al., 2013; Baggs et al., 2017). The majority of R protein NBS-LRRs, such as *A. thaliana* RPS4 and tobacco N., belong to the apoptotic ATPase family of P-loop NTPases. The accumulation of RHIFs possessing a P-loop NTPase domain in plant cells perturbs signal transduction mediated by the R protein. Such perturbance mediated by the P-loop NTPase likely results in ETI induction and symptom development caused by RHIFs.

Motif analysis using PSORT and cNLS Mapper showed that NRHIF localizes mainly to the cytosol. When NRHIF-Venus was expressed in rice cells, Venus-derived yellow fluorescence was observed in both nucleus and cytosol. Since the predicted molecular mass of NRHIF-Venus is 65 kDa, it can diffuse to a limited extent into the nucleus without a nuclear localization signal (NLS). Since NRHIF lacks an NLS, these results suggest that NRHIF localizes mainly to the cytosol in rice cells. The presence of RHIFs in the cytoplasm is reasonable if the enzymatic addition of the 5-carbon isopentenyl moiety to the extracellular amine of adenine 37 by the RHIF P-loop NTPase domain is responsible for inducing HR cell death and the development of symptoms.

Pathogenic bacteria are known to secrete multiple effectors into plant cells during infection. These effectors are involved in inducing ETI, suppressing PTI, and establishing infection. We previously reported that screening a library of transposon-tagged *A. avenae* K1 strains based on suppression of flagellin-triggered H_2_O_2_ generation showed that the suppression activity of 156 transposon-tagged K1 mutants was reduced to the same level as that of *KΔT3SS*. Moreover, sequence analysis of regions flanking the transposons revealed that they were inserted into 68 different genes. In addition, multiple proteins were secreted by strains N1141 and K1 using their T3SS (Kondo et al., 2017; Kawaguchi et al., 2021). The presence of multiple effectors in *A. avenae* strains N1141 and K1 raises the question of whether NRHIF and KRHIF are the only ETI-inducible effectors for rice and finger millet, respectively. ETI responses including HR cell death induction and OsNAC4 expression in *NΔRHIF*-inoculated rice cells were reduced to almost the same extent as that in *NΔT3SS*-inoculated rice cells (Figures 3, 4). Similar results were observed in finger millet inoculated with either *KΔRHIF* or *KΔT3SS* (Figure 6). In addition, our screen employing 6,200 transposon-tagged N1141 mutants covered almost all genes in the genome because the whole-genome sequence of strain N1141 indicates the presence of 4,787 open reading frames. Only RHIF has been identified as an ETI-inducible effector by our transposon-tagged N1141 mutant screen. Taken together, our results suggest that NRHIF is the major effector that causes the ETI responses in rice.

Inoculation tests on rice showed that the growth of strain N1141 was increased by NRHIF deficiency, and that growth was reduced by complementation of *NRHIF* in the *NRHIF*-deficient strain, to the same level as wild-type N1141. Such changes in bacterial growth indicate that NRHIF also functions as an ETI-inducible effector when infecting non-host plants. In contrast, KRHIF-deficient strain K1 did not affect bacterial growth in non-host finger millet. This may be due to differences in the contribution of KRHIF and NRHIF to ETI induction in non-host plants. NRHIF was identified by comprehensive screening of transposon-tagged N1141 mutants while KRHIF was identified based on sequence homology with NRHIF. Therefore, it is unclear whether KRHIF expressed by strain K1 is the only effector involved in ETI induction of finger millet. Multiple effectors apart from KRHIF may be involved in recognizing strain K1 and ETI induction by finger millet. Identification of ETI inducers apart from KRHIF in strain K1 is important for elucidating the molecular mechanism underlying the narrow host range of *A. avenae*.

## Data Availability Statement

The original contributions presented in the study are included in the article/Supplementary Material, further inquiries can be directed to the corresponding author.

## Author Contributions

MN, MK, AS, and F-SC conceived, performed, and designed the experiments. MN, MK, and HH analyzed the data and performed the computational analysis. MN, MK, and F-SC wrote the paper. All authors read and approved the final manuscript.

## Conflict of Interest

The authors declare that the research was conducted in the absence of any commercial or financial relationships that could be construed as a potential conflict of interest.

## Publisher’s Note

All claims expressed in this article are solely those of the authors and do not necessarily represent those of their affiliated organizations, or those of the publisher, the editors and the reviewers. Any product that may be evaluated in this article, or claim that may be made by its manufacturer, is not guaranteed or endorsed by the publisher.
