# Supplementary figures and images for: Novel Effector RHIFs Identified From Acidovorax avenae Strains N1141 and K1 Play Different Roles in Host and Non-host Plants
Source: Front Plant Sci. 2021 Aug 6;12:716738. doi: 10.3389/fpls.2021.716738 (PMC8377416; doi:10.3389/fpls.2021.716738)

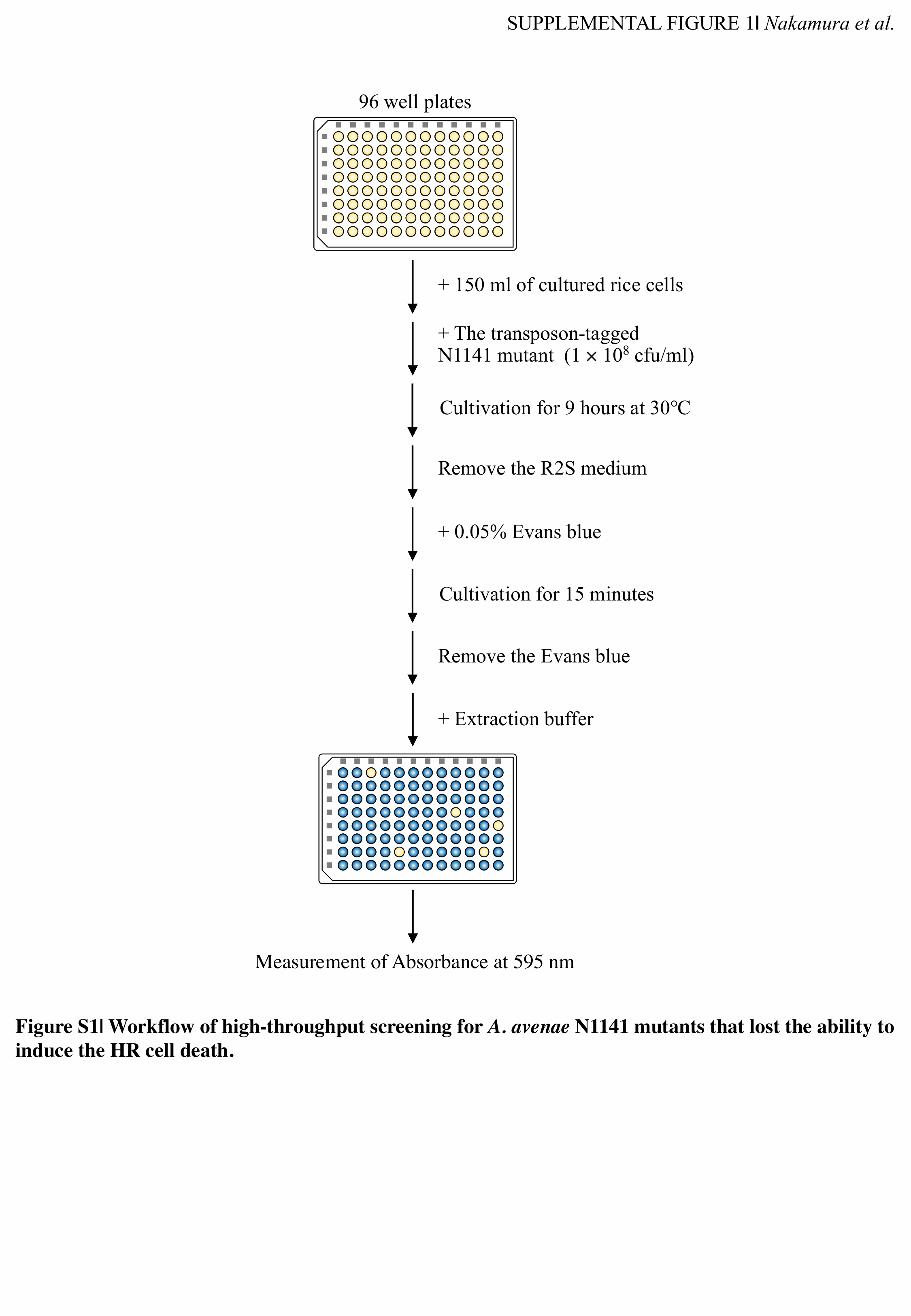

Supplement: Supplementary file 1 [file Image_1.TIF]

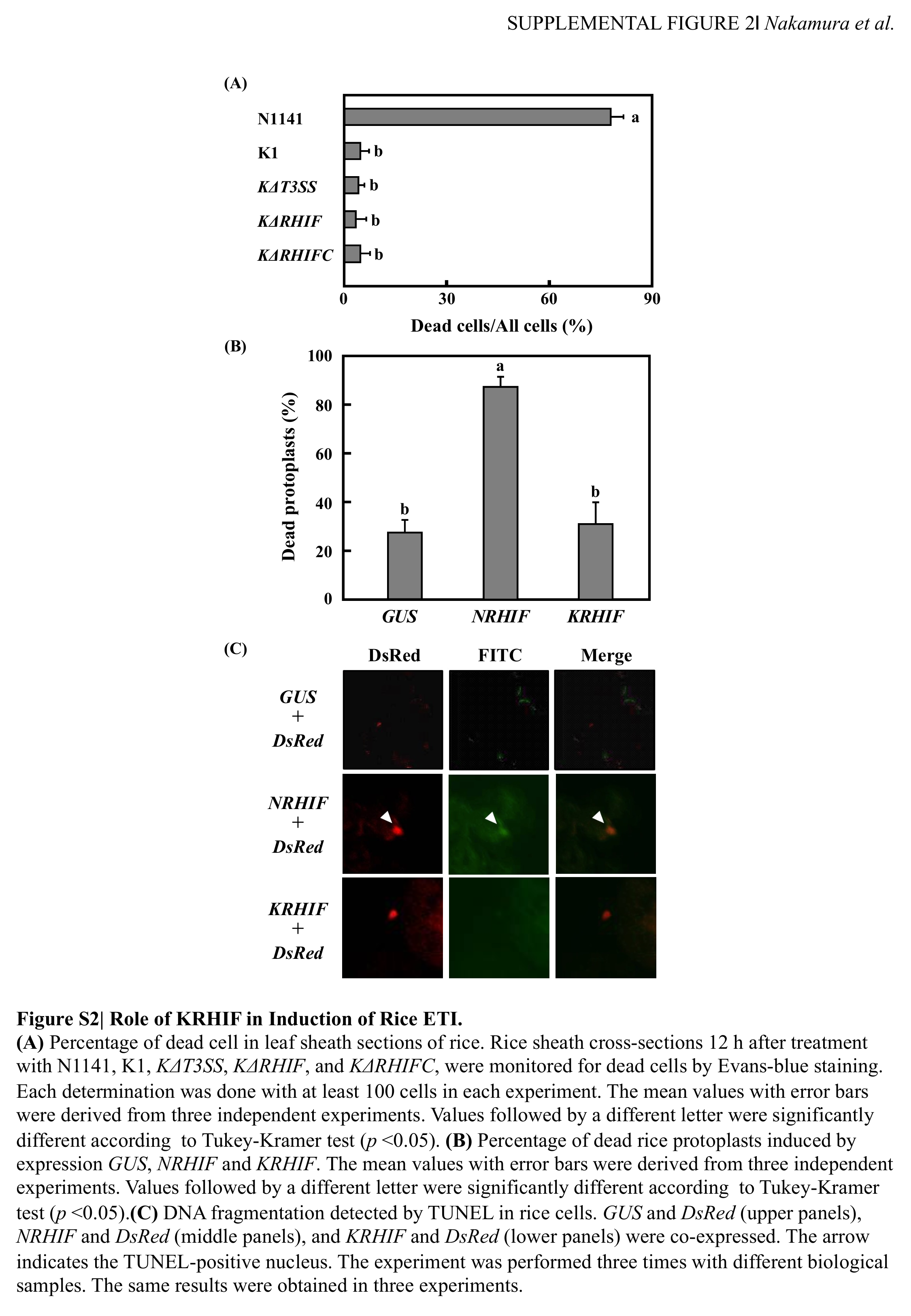

Supplement: Supplementary file 2 [file Image_2.TIF]

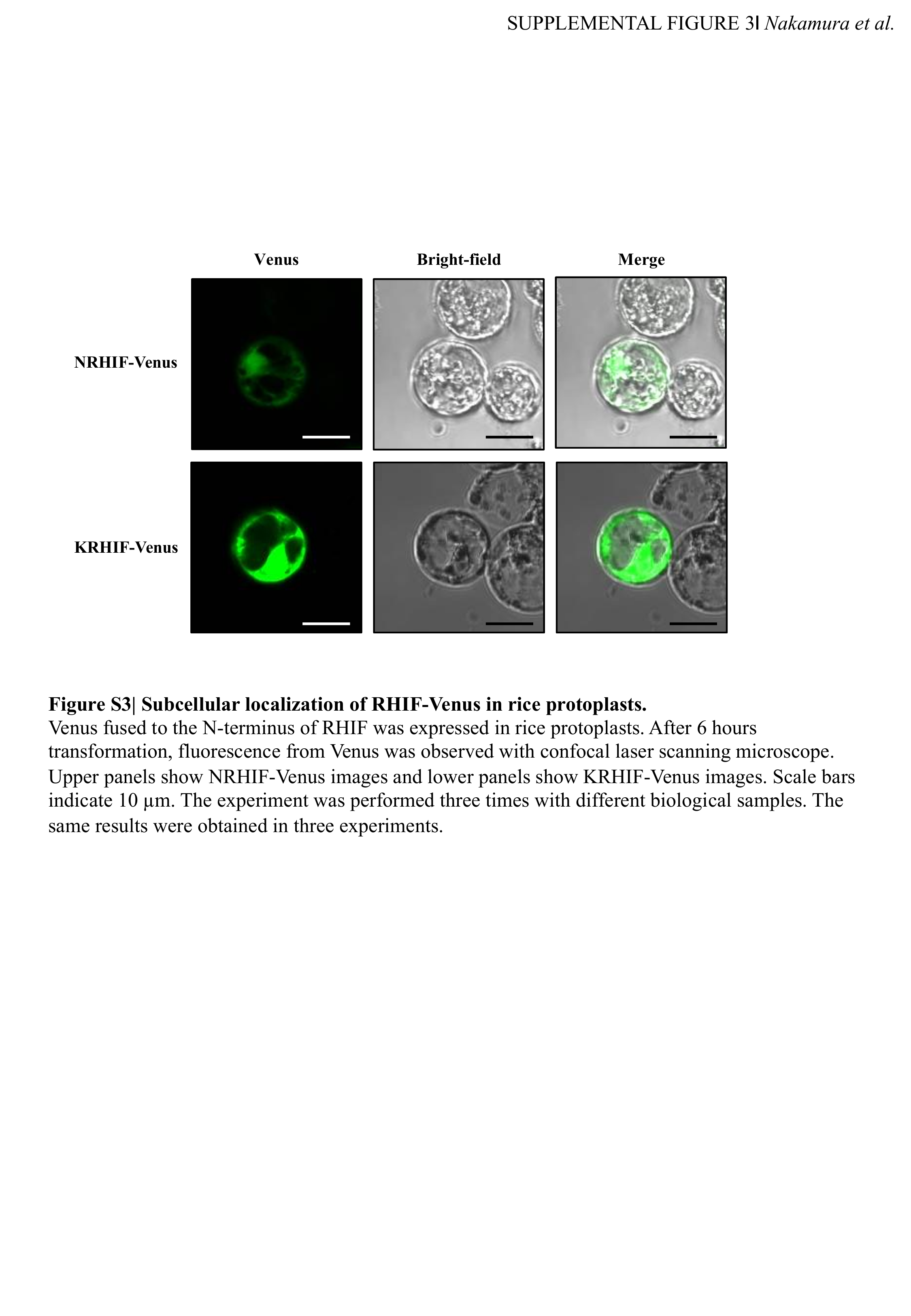

Supplement: Supplementary file 3 [file Image_3.TIF]

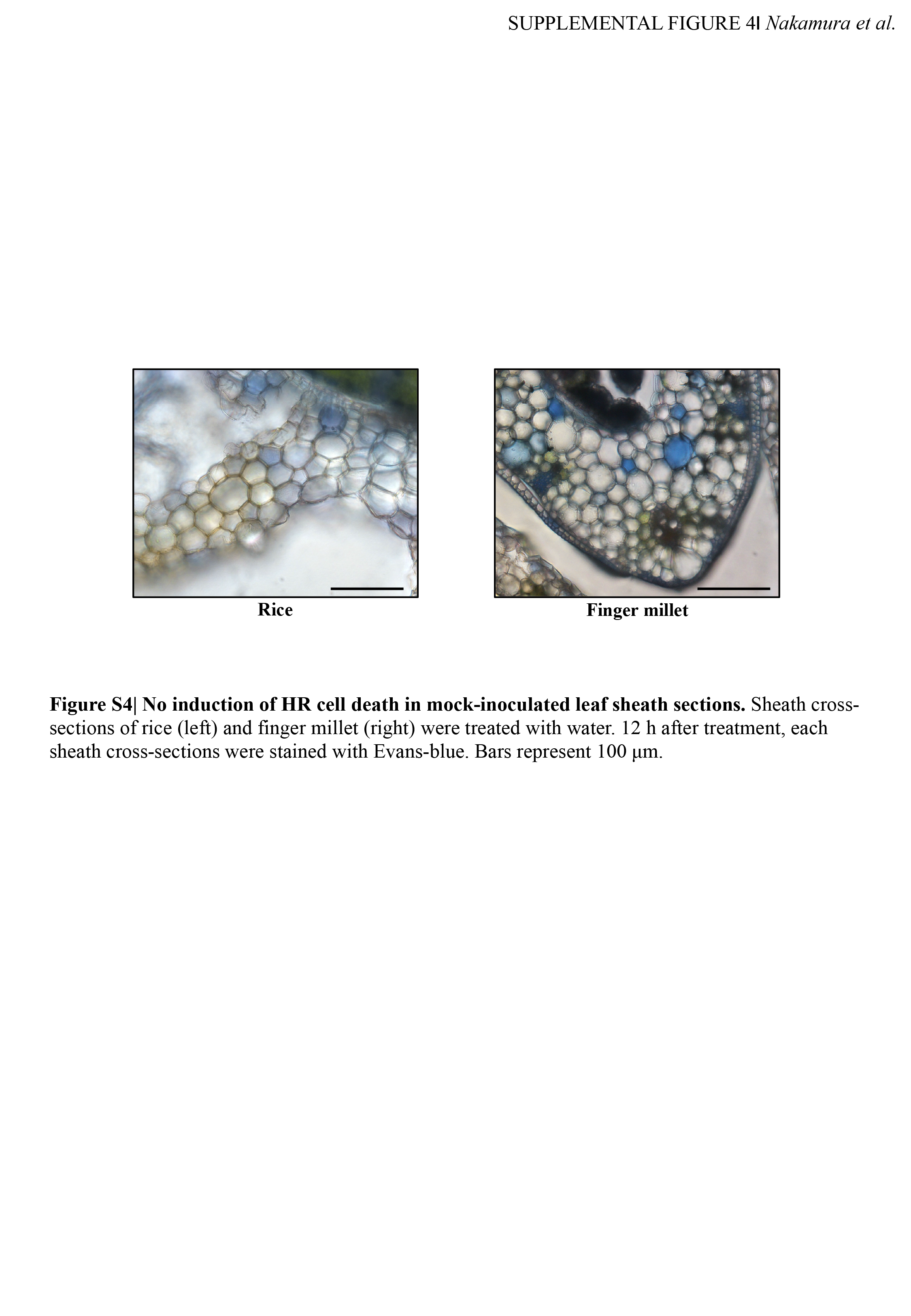

Supplement: Supplementary file 4 [file Image_4.TIF]
